# *Bacillus subtilis* MurJ and Amj Lipid II flippases are not essential for growth

**DOI:** 10.1128/jb.00078-25

**Published:** 2025-04-04

**Authors:** Kiera Englehart, Jonathan Dworkin

**Affiliations:** 1 Department of Microbiology and Immunology, Vagelos College of Physicians and Surgeons, Columbia University 130380 , New York, New York, USA; Geisel School of Medicine at Dartmouth, Hanover, New Hampshire, USA

**Keywords:** peptidoglycan

## Abstract

Identification of the protein that mediates transbilayer transport of the undecaprenyl-pyrophosphate (Und-PP) linked peptidoglycan precursor Lipid II has long been a subject of investigation. Candidates belonging to both the MOP (multidrug/oligosaccharidyl-lipid/polysaccharide) and SEDS (shape, elongation, division and sporulation) families of transmembrane proteins have been proposed, exhibiting characteristics consistent with mediating this process, including genetic essentiality and biochemical activity. While MOP family proteins including MurJ are widely considered to be the primary Lipid II transporter, questions still remain including a role for the SEDS proteins in this process. We and others previously showed that a *Bacillus subtilis* strain lacking all four MurJ homologs is viable, thereby implicating a separate mode of Lipid II transport across the membrane. However, a subsequent report of synthetic essentiality between *B. subtilis* MurJ and the flippase Amj suggested that they are necessary and sufficient. Here, we show that this effect is alleviated by excess synthesis of the enzyme responsible for Und-PP production. Thus, the inviability of a *murJ-amj* double mutant strain is not due to the essentiality of these enzymes for flipping Lipid II but is instead most likely a consequence of a reduction of free Und-PP levels. This result is consistent with a non-MOP-dependent pathway for Lipid II transport across the cytoplasmic membrane to enable cell wall peptidoglycan synthesis.

**IMPORTANCE:**

The assembly of peptidoglycan (PG), the typically essential polymer that provides structural integrity to bacterial cells, begins with the synthesis of the Lipid II monomer in the cytoplasm and along the cytoplasmic face of the inner membrane. Lipid II is then translocated across the membrane to the extracellular site of polymerization. The mechanistic basis for this process remains unclear, with genetic and/or biochemical evidence pointing to two different families of conserved membrane proteins. Here, we present genetic evidence that only one of these two families is essential in *Bacillus subtilis*.

## INTRODUCTION

The mesh-like extracellular peptidoglycan (PG) sacculus provides structural integrity to bacterial cells. As such, PG is typically essential for growth, although under special conditions, bacteria lacking PG can survive. PG is a glycopeptide polymer composed of units of repeating GlcNAc–MurNAc disaccharides covalently attached to stem peptides. The monomeric units are synthesized initially in the cytoplasm and in the final steps, along the cytoplasmic face of the membrane, as a molecule known as Lipid II that contains the glycopeptide attached to a 55-carbon isoprenoid, undecaprenol-pyrophosphate (Und-PP). Lipid II is then transported to the outside of the cell membrane, the site of the transglycosylation (TG) and transpeptidation (TP) activities that assemble the PG polymer (1). These reactions are mediated by two classes of enzymes, the bifunctional class A PBPs, which exhibit both TG and TP activities, and the class B PBPs, which exhibit only TP activity, working in tandem with a SEDS (shape, elongation, division and sporulation) protein with TG activity (2).

The molecular mechanism underlying Lipid II transport remained uncharacterized long after the identification of the enzymes responsible for Lipid II synthesis and polymerization. Integral membrane proteins of the SEDS family are long-hypothesized candidates. The well-studied essential cell division protein SEDS protein FtsW exhibits activity in a fluorescence-based proteoliposome assay consistent with a role in Lipid II transport (3, 4). A second candidate is an essential *Escherichia coli* protein initially known as MviN (5) and subsequently renamed MurJ (6), a member of the MOP (multidrug/oligosaccharidyl-lipid/polysaccharide) family. Experiments using an *in vivo* assay based on the accessibility of translocated Lipid II to cleavage by a degradative enzyme are consistent with a function for MurJ in Lipid II transport in *E. coli* (7). However, *B. subtilis* MurJ and its three homologs are not essential either separately or together (8, 9), raising the question of whether the essential function of MurJ as the Lipid II flippase was phylogenetically conserved. A synthetic lethal screen identified a gene (*amj*) that was synthetically lethal with *B. subtilis murJ* (10). However, Amj has no sequence or predicted structural homology to MurJ (10), so the question remains as to why *B. subtilis* MurJ, unlike *E. coli* MurJ, is not essential. Here, we set out to investigate the origin of this difference.

## RESULTS

**Fig 1:**
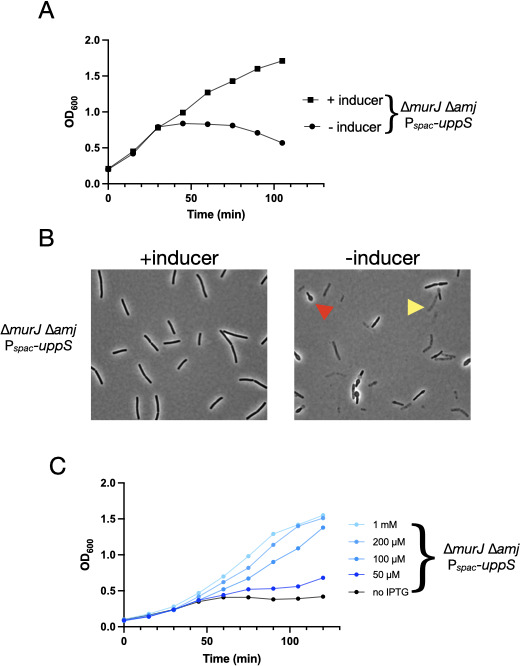
Overexpression of *uppS* restores viability of double mutant ∆*murJ*∆*amj* strain. (A) Strain JDB4540 (∆*murJ ∆amj amyE::*P*_hyspank_–uppS*) was grown in LB in the presence (squares) or absence of 1 mM IPTG (circles) and OD_600_ was recorded. Shown is an example of one of three biological replicates. (B) Samples of the cultures in A obtained at time point t = 90 min were examined by microscopy as described. Lysed (yellow arrow) and morphologically distorted (red arrow) cells are noted. (C) Dose-response rescue of ∆*murJ*∆*amj* strain by *uppS* overexpression. Strain JDB4540 was grown in LB in the absence (black) or the presence of a range of IPTG concentrations (blue hues) and OD_600_ was determined. Shown is one example, and biological replicates exhibited similar patterns of growth.

The original characterization of *E. coli* MurJ (MviN) reported that the growth defect of a *mviN* temperature-sensitive mutation at the non-permissive temperature was suppressed by overexpression of the undecaprenyl pyrophosphate synthase (UppS) (5). This observation suggests that MurJ-associated phenotypes in *B. subtilis* might also be subject to suppression by UppS overexpression. To examine this possibility, we determined if additional Und-PP would affect the phenotype of the *murJ*/*amj* depletion strain. First, we introduced an inducible allele of *uppS* [(P*_hyspank_−uppS*) (11)] into a strain lacking *murJ* alone [∆*murJ::spc*, as derived from the published strain (10)]. Of note, this strain is wildtype with respect to endogenous *uppS*, so induced *uppS* results in levels of Und-PP that are supplemental. We made this strain competent and introduced the *amj* deletion allele (∆*amj::mls*), plating transformants on a selective medium in the presence of an inducer (IPTG). We obtained transformants at a normal transformation frequency and confirmed the presence of the correct *murJ* and *amj* alleles by genome sequencing and backcrossing. Consistent with a requirement for *uppS* expression, colonies were not recovered in the absence of inducer. We then grew one of these transformants (JDB4540; *murJ::spec amyE::*P*_hyspank_-uppS (cat) amj::mls*) in the presence or absence of an inducer, observing that growth was dependent on the presence of an inducer (Fig. 1A). Consistent with the restoration of growth, cells exhibited wild-type morphology in the presence of an inducer including the absence of lysed (yellow) and bulging (red) cells seen without inducer (Fig. 1B). The effect of UppS overexpression on growth is quantitatively dependent on inducer concentration (Fig. 1C), consistent with increased availability of Und-PP mediating the restoration of growth.

We then examined whether the effect of additional UppS expression on the viability of cells lacking MurJ and Amj would be observed under another condition where MurJ homologs are essential for PG synthesis. We investigated the synthesis of the spore cortex PG where the sporulation-specific MurJ homolog *spoVB* is required, and a *spoVB* mutant strain is completely asporogenous (12). We sporulated *spoVB* mutant cells containing an inducible *uppS* allele in the presence or absence of an inducer and monitored sporulation efficiency. As expected, a severe reduction in sporulation was observed in the absence of an inducer. However, the presence of an inducer did not attenuate this defect (Table 1).

**TABLE 1:** Effect of UppS overexpression on the *spoVB* sporulation phenotype^*a*^

Strain	Sporulation
*trpC2*	100%
*∆spoVB*	<10^−5^
*∆spoVB Pspac-uppS* (-IPTG)	<10^−5^
*∆spoVB Pspac-uppS* (+IPTG)	<10^−5^

^*a*^ Shown is one example of three biological replicates that all exhibit similar results.

**Fig 2:**
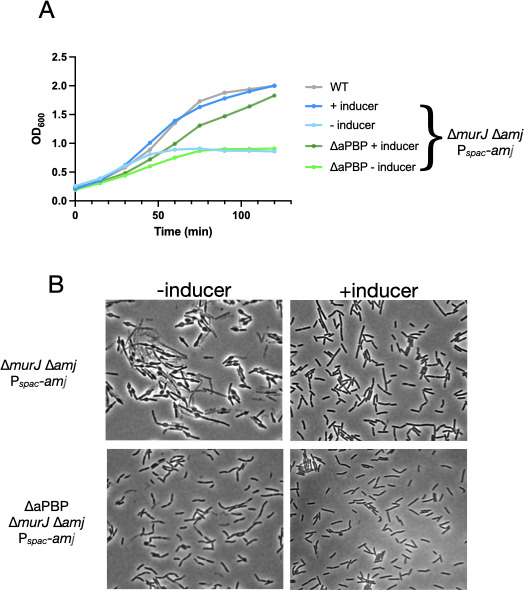
Absence of aPBPs partially suppresses the phenotype of the ∆*murJ*∆*amj* strain. (A) Strain JDB4181 (blue; ∆*murJ* ∆amj Pspank*-amj) and JDB4493 (green; ∆pbpDFG ΔponA ∆murJ ∆amj Pspank*-amj) were grown in LB in the presence or absence of inducer (1 mM IPTG), and OD_600_ was determined. (B) Samples of the cultures in A were examined by microscopy at time point *t* = 120 min as described. Shown is one example, and biological replicates exhibited similar patterns of growth.

Why is Und-PP supplementation successful in rescuing MurJ homolog function during growth but not during sporulation? Of potential relevance to this question is the strict requirement for class A PBP (aPBP) function in sporulation (13) but not *B. subtilis* growth (14, 15). Thus, if aPBP essentiality determines the effectiveness of Und-PP complementation of ∆*murJ* homologs, then aPBPs and MurJ may be functionally related. If so, the absence of aPBP would affect ∆*murJ* phenotypes. To test this hypothesis, we generated a strain lacking all aPBPs [*ponA::kan*; ∆*pbpDFG* (16)] and carrying deletions of both *murJ* and *amj* and an inducible *amj* allele. In the presence of an inducer, both this strain and a strain wildtype for aPBPs grew similarly (Fig. 2A). In the absence of an inducer, both strains exhibited a pronounced decrease in growth rate within 60 min. However, examination of the two strains by light microscopy revealed that the absence of aPBPs substantially mitigated the aberrant morphology of the *murJ/amj* strain observed in the absence of inducer (Fig. 2B). Thus, aPBP activity is functionally associated with *murJ* and *amj*.

## DISCUSSION

We find that the previously reported inviability of a *B. subtilis* strain containing mutations in both *murJ* and *amj* (10) is not due to their synergistic essentiality. We show that supplemental expression of UppS, the cytoplasmic enzyme responsible for the synthesis of Und-PP, allows this strain to grow despite the absence of MurJ and Amj, consistent with a separate system for Lipid II export. Thus, these mutations likely lead to inviability because together they reduce the levels of free Und-PP below that needed for PG synthesis during growth, possibly as a consequence of Und-PP sequestration. This result implies that MurJ and Amj cannot be the sole enzymes mediating the process of Lipid II membrane translocation necessary for PG synthesis during growth in *B. subtilis*.

We further demonstrate that the elimination of aPBP function partially suppresses the phenotype of a *murJ*/*amj* double mutant. This effect suggests that the non-essentiality of *B. subtilis* MurJ is due to the non-essentiality of aPBP function for *B. subtilis* growth. While the loss of aPBP function does alleviate the dramatic morphological pathologies exhibited by cells depleted for *murJ*/*amj*, it does not allow for growth and division. Why this suppression is only partial is not clear, although since a strain lacking aPBPs is viable but phenotypically distinct from the wild-type parent (17), there could be a synergistic interaction between the two classes of mutations. In addition, we observe that UppS supplementation does not suppress the sporulation deficiency of a strain lacking the sporulation-specific MurJ homolog SpoVB. Since PG synthesis in sporulation requires aPBP function (13), UppS-dependent suppression may, therefore, only occur in contexts where aPBP-mediated PG synthesis is not essential. It is formally possible that increased levels of Und-PP affect the activity and/or expression of the other non-sporulation-specific MurJ homolog YabM.

**Fig 3:**
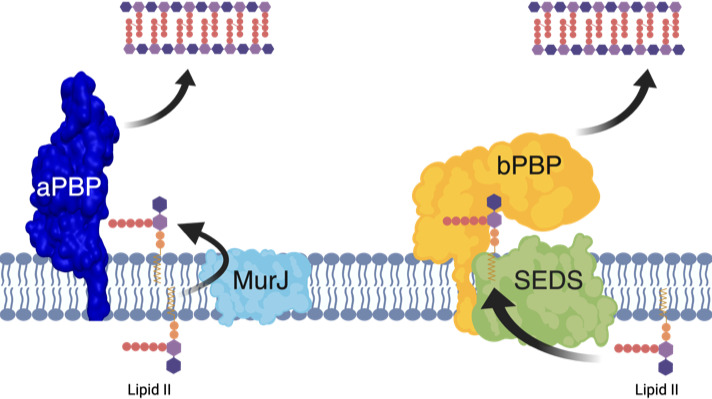
Model of MurJ/Amj function during PG synthesis. Lipid II synthesized on the cytoplasmic face of the membrane is flipped either by MurJ for use by an aPBP (dual TG/TP) or by RodA for use by RodA(TG) and bPBP(TP).

The suppression of morphological defects of the *murJ/amj* mutant strain observed in the absence of aPBPs suggests that MurJ (and possibly Amj) are involved in aPBP-dependent PG synthesis. MurJ is a Lipid II binding protein (18), so it could mediate the transport of cytosolically produced Lipid II used by the aPBPs. (Fig. 3, left). Since growing evidence indicates that aPBP enzymes function separately from the SEDS-family protein complexes containing bPBPs (2, 19), MurJ would then functionally associate with aPBP-containing PG synthetic complexes. This association is likely not mediated by specific protein–protein interactions, as *B. subtilis* Amj can functionally substitute for *E. coli* MurJ, despite the fact that they are not homologs (10).

Our observations could help resolve the apparent paradox of two different proteins—MurJ and FtsW—being identified as Lipid II flippases. *E. coli* MurJ is essential (5, 6) and *murJ* mutations reduce Lipid II accessibility on the extracellular surface of the cytoplasmic membrane (7). *E. coli* FtsW is also essential, and experiments directly monitoring the movement of fluorescent Lipid II across model membranes observed an FtsW-dependence (3, 4). MurJ (20) and FtsW (21) (and its SEDS homolog RodA [22–24]) enzymes have been investigated structurally, but to date, a flippase mechanism has not been definitively established. While MurJ and SEDS proteins could function together in translocating Lipid II (1), an alternative model consistent with the data presented here for *B. subtilis* is that they work with separate PG synthesizing pathways, one containing a SEDS protein (either FtsW or RodA) that flips Lipid II in association with its cognate bPBP and the other including MurJ that flips Lipid II used by an aPBP (Fig. 3).

SEDS proteins have essential TG activity (25), proposed to be coupled to flippase activity (1). Given the intimate nature of the interaction between the TG activity of the SEDS protein and the TP activity of the bPBP (22, 24), it is not clear how Lipid II flipped by a SEDS protein would be made available to aPBPs that are not part of the SEDS-bPBP complex (2, 26). In addition, SEDS proteins likely function as part of a large biosynthetic complex that, as proposed, includes enzymes of the Lipid II synthesis pathway (27, 28). For example, the *E. coli* Lipid II synthase MurG interacts with PBP3, the partner of FtsW (29), and MurG forms a complex with the Lipid I synthase MraY (29) as well as the MurE and MurF enzymes (30). Thus, an appealing aspect of the proposed mechanism is that it would facilitate Lipid II availability for aPBP-dependent PG biosynthesis pathways. That is, MurJ-dependent translocation of Lipid II produced by Mur enzymes functioning independently of the SEDS-bPBP complex would allow both aPBP and bPBP pathways to simultaneously synthesize PG.

The observation reported here regarding *murJ/amj* inviability has parallels in prior work addressing the apparent essentiality of other genes later found to be non-essential. For example, strains carrying knockouts in various teichoic acid biosynthetic genes encoding enzymes active in the later steps of the pathway are not viable, suggesting that teichoic acid is essential (31). However, the deletion of *tagO* that encodes the enzyme mediating the initial step in teichoic acid biosynthesis, the attachment of UDP-GlcNAc to Und-PP, allows for the deletion of later genes in the pathway (32), suggesting that the inviability of *tag* mutant strains is a consequence of Und-PP sequestration away from PG synthesis. In the present context, the alleviation of *murJ*/*amj* synthetic lethality by UppS expression would also result from Und-PP sequestration. While the mechanistic basis of this sequestration is unclear, the phenomenon of Und-PP pools being subject to competition by different cellular processes with PG synthesis is well characterized. For example, enterobacterial O-antigen synthesis also requires Und-PP, and disrupting O-antigen synthesis results in cell shape deformities suppressed by supplemental Und-PP expression (33). Again, the specific mechanism underlying Und-PP sequestration in this context is unclear. Mutation of an enzyme mediating enterobacterial common antigen (ECA) biosynthesis caused defects in cell shape that were suppressed by increasing Und-PP levels. In this case, sequestration was attributed to the accumulation of the Und-PP-1-linked intermediate ECA-lipid II (34).

Tight regulation of Und-PP pools is evolutionarily conserved (35, 36). This may be a consequence of the phenomenon that an overabundance of membrane-associated polyisoprenols, particularly an extended C-55 polyprenol like Und-PP, can disrupt the phospholipid bilayer architecture (37), thereby restricting the size of Und-PP pools. Given this constraint, any Und-PP-consuming pathway must function so as to not interfere with the Und-PP pool required for optimal PG synthesis, a rate-limiting step in bacterial growth (38). Recent work demonstrated the key role of the extracellular sigma factor SigM in maintaining this homeostasis (39). Such metabolic coordination is presumably characteristic of normal physiological contexts and may become particularly relevant to a more pathological context such as a mutation in one of these pathways (34). The observations presented here suggest that mutations affecting a non-essential PG biosynthetic pathway such as that mediated by aPBPs in *B. subtilis* can similarly affect essential bPBP-dependent PG synthesis.

In summary, this work is most consistent with a model for *B. subtilis* PG biosynthesis in which the MurJ and/or Amj Lipid II flippases support normal growth but are not absolutely essential, and SEDS Lipid II flippases participate in essential elongasome and divisome function. Thus, the MOP and SEDS systems may provide a dual route for Lipid II export to the outer face of the cytoplasmic membrane, as postulated (28).

## MATERIALS AND METHODS

### Bacterial growth and sporulation

Strains were grown in Lysogeny Broth (LB) with shaking at 37°C. For measurement of growth curves, an overnight culture from a single freshly streaked colony in the mid-log phase (OD_600_ ~0.3) was diluted 1:10 in 10 mL LB and grown to mid-log. The culture was centrifuged (5′, 3,000 rpm), the pellet was washed in pre-warmed LB, and resuspended in 2 mL LB. One mL was added to a flask containing 9 mL LB and one mL to a flask with 9 mL LB with IPTG as noted. For sporulation, strains were grown in Difco Sporulation Media (DSM) for 24 h at 37°C on a roller-drum, with 1 mM IPTG as noted. Sporulation efficiency was determined by measuring colony-forming units on LB plates before and after heating to 80°C for 15 min and is calculated relative to the efficiency of the wild-type parent.

### Strain construction

Strains were derived from *B. subtilis* 168 *trpC2* and are listed in Table 2. Strains were constructed by transformation using conventional methodology, and where necessary, media was supplemented with 100 µg/mL spectinomycin, 10 µg/mL kanamycin, 5 µg/mL chloramphenicol, and 1× MLS or 20 mM MgCl_2_ (for strains carrying aPBP mutations). JDB4540 was constructed by transforming JDB4005 gDNA into a *murJ::spc* strain (derived from JDB4181), selecting for cm^R^. The resulting strain was transformed with JDB4181 gDNA, selecting for mls^R^ in the presence of 1 mM IPTG. JDB4493 was constructed by transforming JDB4181 gDNA into JDB4482, selecting for spec^R^. This strain was transformed with JDB4181 gDNA selecting for cmR. The resulting strain was transformed with JDB4181 gDNA, selecting for mlsR in the presence of IPTG. Finally, JDB4443 gDNA was transformed into this strain, selecting for kan^R^ in the presence of IPTG. JDB4547 was constructed by transforming JDB4006 gDNA into JDB1097, selecting for cm^R^.

**TABLE 2:** Strains

Strain	Genotype	Source
JDB1772	*trpC2*	Lab collection
JDB1097	*spoVBΔ::tet*	Lab collection
JDB4005	*W168 kan::uppS1 amyE::Pspac(hy)-uppS-FLAG (cat*)	(11)
JDB4181	ycgO::Pspank*-amj(cat) murJ::spec amj::mls sacA::Pveg-mcherry(tet)	(10)
JDB4540	*murJ::spec amyE::*P*_hyspank_−uppS (cat) amj::mls*	This work
JDB4443	*ponA::kan*	*Bacillus subtilis* Genetic Stock Center
JDB4482	*∆pbpDFG*	Helmann lab
JDB4493	∆pbpDFG ytgP::spec ycgO::Pspank*-ydaH(cat) ydaH::mls Δ*ponA::kan*	This work
JDB4547	*spoVBΔ::tet amyE::Pspac(hy)-uppS (cat*)	This work

### Microscopy

Cells were immobilized on 1% agar pads in PBS. Phase contrast microscopy was performed using a Nikon Eclipse 90i microscope and a CFI Plan Apo 100×, NA 1.45 oil objective. Images were taken with a Hamamatsu Orca ER-AG camera and processed with ImageJ.

## References

[1] Egan AJF, Errington J, Vollmer W. 2020. Regulation of peptidoglycan synthesis and remodelling. Nat Rev Microbiol 18:446–460. doi:10.1038/s41579-020-0366-3 32424210

[2] Straume D, Piechowiak KW, Kjos M, Håvarstein LS. 2021. Class A PBPs: it is time to rethink traditional paradigms. Mol Microbiol 116:41–52. doi:10.1111/mmi.14714 33709487

[3] Mohammadi T, van Dam V, Sijbrandi R, Vernet T, Zapun A, Bouhss A, Diepeveen-de Bruin M, Nguyen-Distèche M, de Kruijff B, Breukink E. 2011. Identification of FtsW as a transporter of lipid-linked cell wall precursors across the membrane. EMBO J 30:1425–1432. doi:10.1038/emboj.2011.61 21386816 PMC3102273

[4] Mohammadi T, Sijbrandi R, Lutters M, Verheul J, Martin NI, den Blaauwen T, de Kruijff B, Breukink E. 2014. Specificity of the transport of lipid II by FtsW in Escherichia coli. J Biol Chem 289:14707–14718. doi:10.1074/jbc.M114.557371 24711460 PMC4031526

[5] Inoue A, Murata Y, Takahashi H, Tsuji N, Fujisaki S, Kato J. 2008. Involvement of an essential gene, mviN, in murein synthesis in Escherichia coli. J Bacteriol 190:7298–7301. doi:10.1128/JB.00551-08 18708495 PMC2580715

[6] Ruiz N. 2008. Bioinformatics identification of MurJ (MviN) as the peptidoglycan lipid II flippase in Escherichia coli. Proc Natl Acad Sci USA 105:15553–15557. doi:10.1073/pnas.0808352105 18832143 PMC2563115

[7] Sham LT, Butler EK, Lebar MD, Kahne D, Bernhardt TG, Ruiz N. 2014. Bacterial cell wall. MurJ is the flippase of lipid-linked precursors for peptidoglycan biogenesis. Science 345:220–222. doi:10.1126/science.1254522 25013077 PMC4163187

[8] Fay A, Dworkin J. 2009. Bacillus subtilis homologs of MviN (MurJ), the putative Escherichia coli lipid II flippase, are not essential for growth. J Bacteriol 191:6020–6028. doi:10.1128/JB.00605-09 19666716 PMC2747889

[9] Vasudevan P, McElligott J, Attkisson C, Betteken M, Popham DL. 2009. Homologues of the Bacillus subtilis SpoVB protein are involved in cell wall metabolism. J Bacteriol 191:6012–6019. doi:10.1128/JB.00604-09 19648239 PMC2747891

[10] Meeske AJ, Sham LT, Kimsey H, Koo BM, Gross CA, Bernhardt TG, Rudner DZ. 2015. MurJ and a novel lipid II flippase are required for cell wall biogenesis in Bacillus subtilis. Proc Natl Acad Sci USA 112:6437–6442. doi:10.1073/pnas.1504967112 25918422 PMC4443310

[11] Lee YH, Helmann JD. 2013. Reducing the level of undecaprenyl pyrophosphate synthase has complex effects on susceptibility to cell wall antibiotics. Antimicrob Agents Chemother 57:4267–4275. doi:10.1128/AAC.00794-13 23796923 PMC3754353

[12] Popham DL, Stragier P. 1991. Cloning, characterization, and expression of the spoVB gene of Bacillus subtilis. J Bacteriol 173:7942–7949. doi:10.1128/jb.173.24.7942-7949.1991 1744050 PMC212588

[13] McPherson DC, Driks A, Popham DL. 2001. Two class A high-molecular-weight penicillin-binding proteins of Bacillus subtilis play redundant roles in sporulation. J Bacteriol 183:6046–6053. doi:10.1128/JB.183.20.6046-6053.2001 11567005 PMC99684

[14] Popham DL, Setlow P. 1996. Phenotypes of Bacillus subtilis mutants lacking multiple class A high-molecular-weight penicillin-binding proteins. J Bacteriol 178:2079–2085. doi:10.1128/jb.178.7.2079-2085.1996 8606187 PMC177908

[15] McPherson DC, Popham DL. 2003. Peptidoglycan synthesis in the absence of class A penicillin-binding proteins in Bacillus subtilis. J Bacteriol 185:1423–1431. doi:10.1128/JB.185.4.1423-1431.2003 12562814 PMC142859

[16] Patel Y, Zhao H, Helmann JD. 2020. A regulatory pathway that selectively up-regulates elongasome function in the absence of class A PBPs. Elife 9:e57902. doi:10.7554/eLife.57902 32897856 PMC7478892

[17] Murray T, Popham DL, Setlow P. 1998. Bacillus subtilis cells lacking penicillin-binding protein 1 require increased levels of divalent cations for growth. J Bacteriol 180:4555–4563. doi:10.1128/JB.180.17.4555-4563.1998 9721295 PMC107467

[18] Bolla JR, Sauer JB, Wu D, Mehmood S, Allison TM, Robinson CV. 2018. Direct observation of the influence of cardiolipin and antibiotics on lipid II binding to MurJ. Nature Chem 10:363–371. doi:10.1038/nchem.2919 29461535 PMC5912511

[19] Cho H, Wivagg CN, Kapoor M, Barry Z, Rohs PDA, Suh H, Marto JA, Garner EC, Bernhardt TG. 2016. Bacterial cell wall biogenesis is mediated by SEDS and PBP polymerase families functioning semi-autonomously. Nat Microbiol 1:16172. doi:10.1038/nmicrobiol.2016.172 27643381 PMC5030067

[20] Kuk ACY, Mashalidis EH, Lee S-Y. 2017. Crystal structure of the MOP flippase MurJ in an inward-facing conformation. Nat Struct Mol Biol 24:171–176. doi:10.1038/nsmb.3346 28024149 PMC5382020

[21] Käshammer L, van den Ent F, Jeffery M, Jean NL, Hale VL, Löwe J. 2023. Cryo-EM structure of the bacterial divisome core complex and antibiotic target FtsWIQBL. Nat Microbiol 8:1149–1159. doi:10.1038/s41564-023-01368-0 37127704 PMC7614612

[22] Sjodt M, Rohs PDA, Gilman MSA, Erlandson SC, Zheng S, Green AG, Brock KP, Taguchi A, Kahne D, Walker S, Marks DS, Rudner DZ, Bernhardt TG, Kruse AC. 2020. Structural coordination of polymerization and crosslinking by a SEDS-bPBP peptidoglycan synthase complex. Nat Microbiol 5:813–820. doi:10.1038/s41564-020-0687-z 32152588 PMC7540724

[23] Sjodt M, Brock K, Dobihal G, Rohs PDA, Green AG, Hopf TA, Meeske AJ, Srisuknimit V, Kahne D, Walker S, Marks DS, Bernhardt TG, Rudner DZ, Kruse AC. 2018. Structure of the peptidoglycan polymerase RodA resolved by evolutionary coupling analysis. Nature 556:118–121. doi:10.1038/nature25985 29590088 PMC6035859

[24] Nygaard R, Graham CLB, Belcher Dufrisne M, Colburn JD, Pepe J, Hydorn MA, Corradi S, Brown CM, Ashraf KU, Vickery ON, Briggs NS, Deering JJ, Kloss B, Botta B, Clarke OB, Columbus L, Dworkin J, Stansfeld PJ, Roper DI, Mancia F. 2023. Structural basis of peptidoglycan synthesis by E. coli RodA-PBP2 complex. Nat Commun 14:5151. doi:10.1038/s41467-023-40483-8 37620344 PMC10449877

[25] Meeske AJ, Riley EP, Robins WP, Uehara T, Mekalanos JJ, Kahne D, Walker S, Kruse AC, Bernhardt TG, Rudner DZ. 2016. SEDS proteins are a widespread family of bacterial cell wall polymerases. Nature 537:634–638. doi:10.1038/nature19331 27525505 PMC5161649

[26] Straume D, Piechowiak KW, Olsen S, Stamsås GA, Berg KH, Kjos M, Heggenhougen MV, Alcorlo M, Hermoso JA, Håvarstein LS. 2020. Class A PBPs have A distinct and unique role in the construction of the pneumococcal cell wall. Proc Natl Acad Sci USA 117:6129–6138. doi:10.1073/pnas.1917820117 32123104 PMC7084106

[27] Graham CLB, Newman H, Gillett FN, Smart K, Briggs N, Banzhaf M, Roper DI. 2021. Dynamic network of proteins facilitate cell envelope biogenesis in gram-negative bacteria. Int J Mol Sci 22:12831. doi:10.3390/ijms222312831 34884635 PMC8657477

[28] Walter A, Mayer C. 2019. Peptidoglycan structure, biosynthesis, and dynamics during bacterial growth. In Cohen E, Merzendorfer H (ed), Extracellular Sugar-Based Biopolymers Matricies. Springer Nature, Switzerland.

[29] Mohammadi T, Karczmarek A, Crouvoisier M, Bouhss A, Mengin-Lecreulx D, den Blaauwen T. 2007. The essential peptidoglycan glycosyltransferase MurG forms a complex with proteins involved in lateral envelope growth as well as with proteins involved in cell division in Escherichia coli. Mol Microbiol 65:1106–1121. doi:10.1111/j.1365-2958.2007.05851.x 17640276 PMC2170320

[30] Laddomada F, Miyachiro MM, Jessop M, Patin D, Job V, Mengin-Lecreulx D, Le Roy A, Ebel C, Breyton C, Gutsche I, Dessen A. 2019. The MurG glycosyltransferase provides an oligomeric scaffold for the cytoplasmic steps of peptidoglycan biosynthesis in the human pathogen Bordetella pertussis. Sci Rep 9:4656. doi:10.1038/s41598-019-40966-z 30874582 PMC6420597

[31] Bhavsar AP, Erdman LK, Schertzer JW, Brown ED. 2004. Teichoic acid is an essential polymer in Bacillus subtilis that is functionally distinct from teichuronic acid. J Bacteriol 186:7865–7873. doi:10.1128/JB.186.23.7865-7873.2004 15547257 PMC529093

[32] D’Elia MA, Millar KE, Beveridge TJ, Brown ED. 2006. Wall teichoic acid polymers are dispensable for cell viability in Bacillus subtilis. J Bacteriol 188:8313–8316. doi:10.1128/JB.01336-06 17012386 PMC1698200

[33] Jorgenson MA, Young KD. 2016. Interrupting biosynthesis of O antigen or the lipopolysaccharide core produces morphological defects in Escherichia coli by sequestering undecaprenyl phosphate. J Bacteriol 198:3070–3079. doi:10.1128/JB.00550-16 27573014 PMC5075036

[34] Jorgenson MA, Kannan S, Laubacher ME, Young KD. 2016. Dead-end intermediates in the enterobacterial common antigen pathway induce morphological defects in Escherichia coli by competing for undecaprenyl phosphate. Mol Microbiol 100:1–14. doi:10.1111/mmi.13284 26593043 PMC4845916

[35] Barreteau H, Magnet S, El Ghachi M, Touzé T, Arthur M, Mengin-Lecreulx D, Blanot D. 2009. Quantitative high-performance liquid chromatography analysis of the pool levels of undecaprenyl phosphate and its derivatives in bacterial membranes. J Chromatogr B Analyt Technol Biomed Life Sci 877:213–220. doi:10.1016/j.jchromb.2008.12.010 19110475

[36] Workman SD, Strynadka NCJ. 2020. A slippery scaffold: synthesis and recycling of the bacterial cell wall carrier lipid. J Mol Biol 432:4964–4982. doi:10.1016/j.jmb.2020.03.025 32234311

[37] Hartley MD, Imperiali B. 2012. At the membrane frontier: a prospectus on the remarkable evolutionary conservation of polyprenols and polyprenyl-phosphates. Arch Biochem Biophys 517:83–97. doi:10.1016/j.abb.2011.10.018 22093697 PMC3253937

[38] Belliveau NM, Chure G, Hueschen CL, Garcia HG, Kondev J, Fisher DS, Theriot JA, Phillips R. 2021. Fundamental limits on the rate of bacterial growth and their influence on proteomic composition. Cell Syst 12:924–944. doi:10.1016/j.cels.2021.06.002 34214468 PMC8460600

[39] Roney IJ, Rudner DZ. 2024. Bacillus subtilis uses the SigM signaling pathway to prioritize the use of its lipid carrier for cell wall synthesis. PLoS Biol 22:e3002589. doi:10.1371/journal.pbio.3002589 38683856 PMC11081497

